# Impact of Intensive Care Unit Readmissions on Patient Outcomes and the Evaluation of the National Early Warning Score to Prevent Readmissions: Literature Review

**DOI:** 10.2196/13782

**Published:** 2020-05-08

**Authors:** Heidi Mcneill, Saif Khairat

**Affiliations:** 1 School of Nursing University of North Carolina at Chapel Hill Chapel Hill, NC United States; 2 Carolina Health Informatics Program University of North Carolina at Chapel Hill Chapel Hill, NC United States

**Keywords:** patients, critical care, hospitalization, risk, news

## Abstract

**Background:**

Intensive care unit (ICU) readmissions have been shown to increase a patient’s in-hospital mortality and length of stay (LOS). Despite this, no methods have been set in place to prevent readmissions from occurring.

**Objective:**

The aim of this literature review was to evaluate the impact of ICU readmission on patient outcomes and to evaluate the effect of using a risk stratification tool, the National Early Warning Score (NEWS), on ICU readmissions.

**Methods:**

A database search was performed on PubMed, Cumulative Index of Nursing and Allied Health Literature, Google Scholar, and ProQuest. In the initial search, 2028 articles were retrieved; after inclusion and exclusion criteria were applied, 12 articles were ultimately used in this literature review.

**Results:**

This literature review found that patients readmitted to the ICU have an increased mortality rate and LOS at the hospital. The sample sizes in the reviewed studies ranged from 158 to 745,187 patients. Readmissions were most commonly associated with respiratory issues about 18% to 59% of the time. The NEWS has been shown to detect early clinical deterioration in a patient within 24 hours of transfer, with a 95% CI of 0.89 to 0.94 (*P*<.001), a sensitivity of 93.6% , and a specificity of 82.2%.

**Conclusions:**

ICU readmissions are associated with worse patient outcomes, including hospital mortality and increased LOS. Without the use of an objective screening tool, the provider has been solely responsible for the decision of patient transfer. Assessment with the NEWS could be helpful in decreasing the frequency of inappropriate transfers and ultimately ICU readmission.

## Introduction

### Background

There is a problem associated with patients being readmitted to the intensive care unit (ICU) following transfer from the ICU to the medical floor. Hospitals rely on quality metrics to address many aspects of patient care; one of those metrics is the unplanned ICU readmission within 72 hours of transfer [1]. ICU readmission or *bounce backs* are associated with worse outcomes for the patient and increased resource utilization [2]. The poor outcomes include increased hospital mortality [3]; increased length of stay (LOS) [4], which ultimately effects the availability of ICU beds [2]; and increased hospital costs [5]. An increase in hospital cost not only affects the patient but also impacts the entire hospital system, as the ICU readmission rate is associated with the performance of the ICU and the hospital [6-8].

Unexpected readmission to the ICU is associated with significantly high hospital mortality compared with patients who are not readmitted: 21.3% to 40% compared with 3.6% to 8.4% [9-17]. The odds of death remain 6 to 7 times higher among readmitted patients, independent of other factors [11]. Among the most common reasons for readmissions were pneumonia and respiratory failure [18,19]. Although primary reasons for readmission to the ICU have been established within the medical critical care population, the reasons have not clearly been delineated for the surgical population [1].

This problem has been identified at the national level [1,5] and at the local level as well. Surgical trauma ICU readmissions have been noted specifically at a local hospital in the southern Piedmont region of North Carolina. The major predictor of surgical trauma ICU readmissions within this organization is respiratory failure. A goal has been set within this organization to identify the patients at high risk for bouncing back to the surgical trauma ICU after transfer and to prevent these bounce backs from occurring. The use of a risk stratification tool, such as the National Early Warning Score (NEWS), could identify the patients at high risk for readmission to the surgical trauma ICU and, therefore, prevent their premature transfer to the progressive care or medical units [20].

### Objective

The aim of this literature review was to evaluate the impact of ICU readmission on patient outcomes and to evaluate the effect of using a risk stratification tool, the NEWS, on ICU readmissions.

## Methods

### Search Strategy

In a review of the literature, focus was placed on ICU readmissions and assessment tools utilized for patient transfers. The literature was evaluated to answer the 2 proposed questions. The first question was as follows: Does ICU readmission increase mortality and LOS? If so, what are the readmission rate and risk factors for readmission? The second question was as follows: Is the NEWS an adequate tool for evaluating the patient’s readiness for transfer out of the ICU to the medical floor? A search was conducted on the electronic databases PubMed, Cumulative Index of Nursing and Allied Health Literature, Google Scholar, and ProQuest. The search was limited to articles that were published within the last 10 years, ie, the publication date had to be 2008 onward. Key search terms were ICU, critical care, surgical, and assessment tool. Search terms were identified in the abstract of other articles, and these were used to expand the search. Additional key terms used included “characteristics,” “readmission, risk,” “trauma,” and “National Early Warning Score” (NEWS). Medical Subject Headings terms included “(ICU readmission AND risk), ((ICU readmission AND risk) AND (assessment tool))”, and “(ICU AND (bouncebacks OR bounce backs) AND trauma).”

### Inclusion and Exclusion Criteria

In addition to searching key terms, the bibliographies of the articles reviewed were also searched, and key articles were identified that were useful in the evaluation of ICU readmissions and assessment tools. The articles were independently searched and evaluated for use within the review of the literature. Of the 519 articles identified, the top 200 articles were screened for review. The inclusion criteria for the search were studies of adults, readmissions to the ICU, and articles that focused on the NEWS as a screening tool. Of the 200 articles identified, the titles and abstracts were reviewed, and 13 articles were considered to have met the inclusion criteria. The exclusion criteria were studies that were not published in the English language, studies that were published before 2008, studies that did not include full-text articles, and studies that reviewed other risk assessment tools such as the Minimizing ICU Readmission score. Of note, one article was reviewed as it evaluated the Stability and Workload Index for Transfer score because it provided details on readmission and mortality rates in the surgical patient population [21]. A literature matrix has been provided to break down the articles that were reviewed. Of those articles, there were 2 systematic reviews, 5 retrospective chart reviews, 1 descriptive study, and 4 prospective studies.

## Results

### Evaluation

A thorough review of the literature was performed to identify the risk factors associated with ICU readmission and to examine if the NEWS is an adequate tool for evaluating the patient’s readiness for transfer out of the ICU. After inclusion and exclusion criteria were applied, a total of 200 articles were reviewed, and 13 articles were utilized in this literature review. A literature matrix has been included within Multimedia Appendix 1, outlining the articles reviewed. The evidence level of all articles has been identified within the literature matrix. Additionally, the findings of each article can be referenced in Multimedia Appendix 2. Among the articles reviewed, the most popular research method was a prospective observational study. Of the 13 articles that were reviewed, it is important to note that 2 were systematic reviews and represented the highest level of evidence. The sample sizes within the studies ranged from 158 to 745,187 patients [5,22]. The definition of bounce back ranged in the studies from 72 hours to 7 days [2,9,21,23].

### Readmission Rate

**Figure 1:**
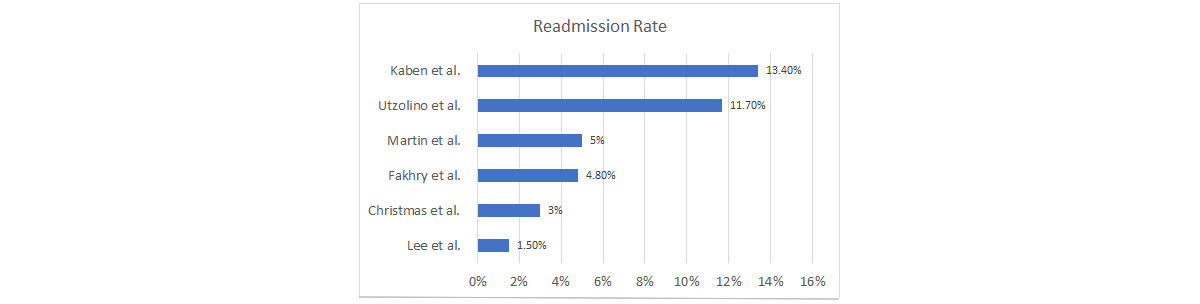
Readmission rate among studies.

The readmission rate of patients transferred out of the ICU to the medical floor was reviewed in 13 articles. Within these articles, the readmission criteria varied; readmissions were considered between 72 hours [2,21,23] and up to 7 days [9]. The readmission rate varied among articles from as low as 1.5% (378/25,717 patients) [23] to as high as 13.4% (381/2852 patients) [9] (Figure 1). The variation in the rates is likely attributed to the difference in readmission time, with Kaben et al [9] allowing 7 days and Lee et al [23] only including readmissions within 3 days. Not only does the readmission time affect the patient’s readmission rate, but other factors such as mental status, age, and sex also play a part in the readmission rate; these factors are discussed further later. It is interesting to note that 1828 out of 2852 (64.1%) patients discharged from the ICU in the study by Kaben et al [9] were men, which was later noted to be a risk factor for readmission. In addition, the patients within this study were strictly monitored for readmission to a surgical ICU setting, whereas the other studies looked at medical and surgical patients, which too could have affected the readmission rate. It is important to note that readmission to the ICU was significantly increased to 25.1% (110/439 patients) in patients with unplanned discharges, that is, those patients who were transferred because of a lack of availability of ICU beds [14]. It is clear from the readmission rates provided within the reviewed articles that allowing a longer period for ICU readmission will increase the readmission rate. It is not clear within the articles which timeframe is most appropriate to deem the patient’s as a true readmission because of inappropriate ICU transfer. It is most likely that identifiable factors for readmission at the time of transfer would show themselves within the first 72 hours of transfer, resulting in readmission. It is not favorable to think that readmissions occurring after 7 days would have shown signs for potential readmission at the time of transfer from the ICU.

### Readmission Risk Factors

**Figure 2:**
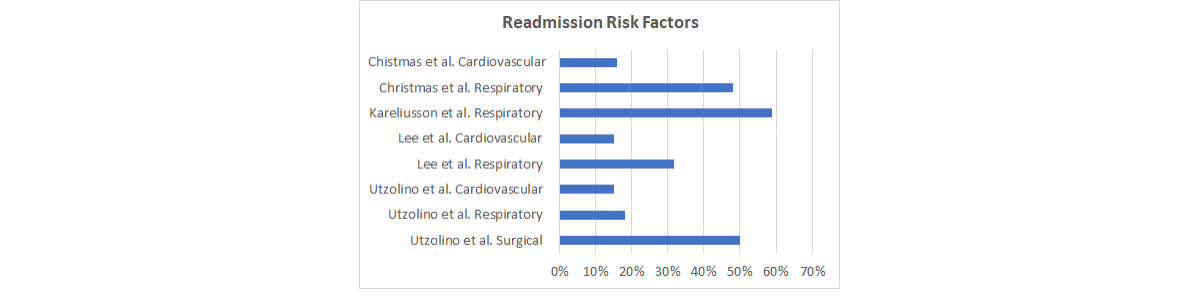
Readmission risk factors among studies.

Although many risk factors were associated with ICU readmission, the most common cause for ICU readmission was respiratory insufficiency or failure, accounting for 18% to 59% of all readmitted patients [14,21]. A total of 72 out of 148 patients were readmitted because of respiratory distress, with 31% requiring intubation [5]. In addition, it was shown that if mechanical ventilation was required on readmission to the ICU, an increased mortality was identified (*P*<.001) [23]. In the case of patients readmitted because of a respiratory failure, it would be important to identify their oxygen requirements before they were discharged from the ICU and if they were receiving any preventative respiratory therapies. The second most common cause of ICU readmission was cardiac etiology; this accounted for 15% to 30.2% of patients readmitted, with one study identifying 91 out of 378 patients readmitted because of cardiovascular issues [14,23]. Figure 2 breaks down the risk factors associated with readmission per study; the figure compares respiratory problems with cardiovascular problems. In additional, Utzolino et al [14] report surgical complications as an additional risk factor for readmission.

Among the studies reviewed, there were additional risk factors that identified a patient to be at a higher risk for ICU readmission, namely, male sex, age greater than 54 years, surgical patient, decreased Glasgow Coma Scale (GCS) at the time of initial transfer from the ICU to the medical unit, and multiple comorbidities at the time of initial transfer [2,5-9,14,21,23,24]. A male patient had an odds ratio of 2.9, and patients with 3 or more comorbidities showed an odds ratio of 8.4, with *P*<.001 for readmission [2]. In addition, according to Christmas et al [5], patients with traumatic brain injury were more vulnerable to ICU readmission; this likely ties into the patient’s GCS at the time of discharge from ICU, with additional research providing an odds ratio of 22.3 and *P*<.01 for the risk of readmission if the patient’s GCS was less than 9 at the time of transfer [2]. There were tools available for screening patients with multiple comorbidities and the risk associated with these diagnoses. These tools were used in the evaluation of comorbidities throughout the literature that was reviewed; however, those tools were not evaluated within this literature review.

### Mortality and Length of Stay

**Figure 3:**
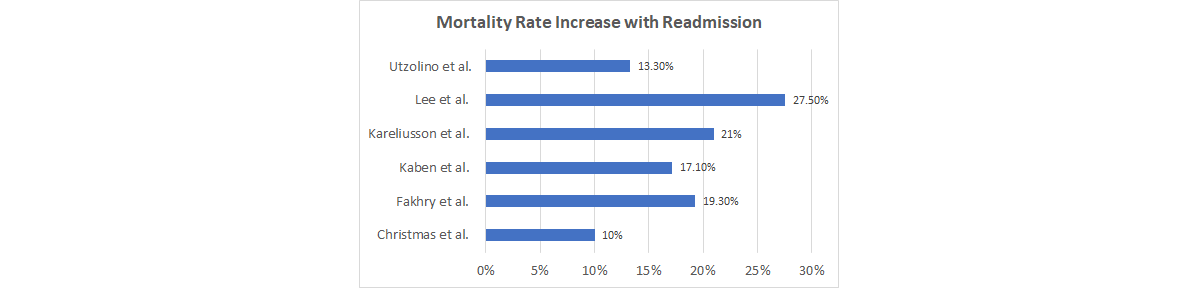
Increase in mortality rate with readmission.

 Although an increased LOS was reported among the studies, the increase was not always quantified; however, it did range from an increase of 11 to 40 days following ICU readmission [5,14,21]. Readmission to the ICU during the patient’s hospitalization ultimately increases the patient’s overall LOS in the hospital. A prolonged ICU admission would ultimately result in a prolonged hospital stay, limiting the number of hospital beds available for new admissions. In addition, all studies noted an increase in mortality rate among patients readmitted to the ICU; mortality ranged from 10% to 27.5% [5,23]. Figure 3 shows the mortality rate in percentage per study. The increase in mortality rate following ICU readmission is likely associated with the severity of the patient’s illness prompting the return to the ICU. According to Utzolino et al [14], the mortality risk was increased if the ICU readmission was related to a respiratory cause; the study additionally showed that 33 out of 249 (13.2%) patients died following their readmission to the ICU. Those patients who were readmitted to the ICU and required mechanical ventilation at that time had an increase in mortality rate, with *P*<.001 [23]. On the basis of these data, those patients who are readmitted to the ICU because of a respiratory issue, especially those requiring mechanical ventilation, will have an increased LOS as well as an increase in their mortality; in addition, those with a cardiac event have the next highest mortality rate.

## Discussion

### Principal Findings

A review of the literature showed that ICU readmission ranged from 1.5% to 13.4% [5,7]. The greatest risk factors identified for ICU readmission include increased age, the male sex, a decreased GCS (less than 9), and multiple comorbidities [2,9,14,21,23,24]. The most common reason for ICU readmission was respiratory distress or failure, with those patients who required mechanical ventilation at the time of readmission showing a greater mortality [23]. Overall, readmission to the ICU increased the patient’s LOS and overall mortality rate from 10% to 27.5% [5,23]. The transition of patient care from the medical ICU to the medical unit is a routine process that exposes patients to preventable adverse events [22]. This transition is often challenging as the sickest patients within the hospital are transferred from a resource-intensive environment to a resource-limited environment [22]. Evidence suggests that readmissions to the ICU, no matter the type, leads to worse patient outcomes. The quality of evidence varied greatly from expert opinion to systematic reviews. Throughout the literature, the parameters quantifying ICU readmission varied from 72 hours to 7 days [2,9,21,23]. The readmission rate varied across the studies from 1.5% to 13.4% [9,23]. Although timing was a factor in the readmission rates, other risk factors for readmission were identified, such as mental status, sex, and age; the variation in patient’s reason to transfer likely also contributed to the variation in readmission rate. An additional driving factor associated with patients being discharged too early from the surgical trauma or medical ICU is the demand for ICU beds [5,14,25]. The demand for ICU beds in the hospital setting is going to remain an issue; however, medical units need to be prepared to provide the level of care that is required by this patient population. Given this demand, ICU readmission rates are a quality metric for hospital care [1,24]. It should also be noted that ICU readmissions ultimately result in increased hospital costs [2].

Kaben et al [9] have shown that patients readmitted to the ICU have an increased incidence of inpatient morbidity and mortality. The evidence of leading factors for ICU readmission varied across the studies reviewed and were contingent on the type of patient being evaluated. For example, it was most commonly noted that patients with respiratory failure [5,21,23]; vital sign instability [2]; and surgical complications, which included anastomotic leak, surgical site infection, and bleeding [14], were most commonly readmitted to the ICU. It has been noted that patients discharged from the ICU with residual organ dysfunction were more likely to be readmitted to the ICU than patients without residual organ dysfunction [23]. The mortality rate was not quantified in every study, but in those in which it was stated, the rate ranged from 10% to 17.1% [5,9]. Given this increased mortality rate associated with ICU readmissions, a risk stratification tool could provide clinicians with the information needed to make an informed decision related to patient transfers. An ideal tool for implementation will forecast patient outcomes and, therefore, facilitate the delivery for safe, effective, and efficient care [22]. Rapid deterioration in patients’ status can occur during their hospitalization because of disease progression; evidence suggests that the signs of deterioration can be identified up to hours of being a serious clinical event [26]. It is, therefore, important for the clinician to make an informed decision about those patients who are appropriate for transfer from the ICU; the use of a risk stratification tool could be helpful in identifying patients at high risk for readmission to the ICU [22]. Screening tools can be effective in alerting clinicians in real time of those high-risk patients and can assist in individualized decision making for their patients [26]. The NEWS is a risk stratification tool and has been evaluated for many clinical outcomes including cardiac arrest, unanticipated ICU admission, or death within 24 hours of admission [20]. For the purpose of this literature review, the NEWS was the only screening tool evaluated. This is a simple tool and can easily be implemented for evaluation of patients before transferring them out of the ICU; it is an aggregated weighted score based on the measurement of 6 vital signs and the level of inspired oxygen [25]. This tool is favored as it has been shown to improve patient outcomes in a variety of settings. It was noted to have identified deterioration in nonelective surgical patients and medical patients [27]. When evaluating patients using NEWS, a higher score indicates a greater severity of illness and an increased risk of adverse events. When patients were evaluated with NEWS before ICU discharge, the scores were an independent predictor of the clinical deterioration of the patients within 24 hours of transfer, with *P*<.001 [25]. This study demonstrated significant sensitivity and specificity in the prediction of clinical deterioration within 24 hours of transfer. The evidence to support the implementation of NEWS was gathered from 3 systematic reviews and 1 prospective observational study. The implementation of the NEWS or any risk stratification tool relies heavily on the use of the tool by clinicians [22]. Therefore, it is important to identify a tool that is easy to use, does not increase the clinician’s or nurse’s workload, and identifies high-risk patients. The NEWS meets all these criteria [27].

### Limitations

Limitations were noted in this review of the literature. The focus of this literature review was to identify the impact of ICU readmission on patient outcomes and to evaluate the NEWS as a tool for assessing patients before transferring them out of the ICU, which would be appropriate for application for surgical trauma critical care patients. Many of the studies identified were performed on medical ICU patients. In addition, the definition of ICU readmission varied among studies, making it difficult to compare the studies that were utilized in this review of the literature. Finally, the study was limited by the strength of the data identified; although 2 systematic reviews were utilized, the remaining studies were prospective and retrospective in nature.

### Conclusions

The evidence reviewed supports the fact that premature transfer from the ICU to the medical floor is associated with adverse outcomes for the patient. Those adverse outcomes affect the patient’s mortality, hospital cost, and the LOS at the hospital. It is important to note that the adverse outcomes are likely not solely because of premature transfer but could be the result of the patient’s overall clinical picture and severity of illness. To this point, ICU transfer has solely been the clinical decision of the provider caring for the patient. This decision is often clouded by the constant need for ICU beds and the request for transfers in the middle of the night to make beds available. A risk stratification tool, such as the NEWS, supplies the provider with objective data to support the decision to transfer the patient and to identify the patients at high risk for readmission. Implementation of a risk stratification tool such as the NEWS would be beneficial in evaluating the patient’s readiness to get transferred from the ICU, in addition to the clinician’s judgment. However, further research needs to focus on the application of the NEWS on the surgical trauma critical care patient.

## Appendix

Multimedia Appendix 1 Preferred Reporting Items for Systematic Reviews and Meta-Analyses flow diagram.

Multimedia Appendix 2 Article tables.

## Abbreviations

GCS: Glasgow Coma Scale
ICU: intensive care unit
LOS: length of stay
NEWS: National Early Warning Score
