# Supplementary material for: Impact of Intensive Care Unit Readmissions on Patient Outcomes and the Evaluation of the National Early Warning Score to Prevent Readmissions: Literature Review
Source: JMIR Perioper Med. 2020 May 8;3(1):e13782. doi: 10.2196/13782 (PMC7709858; doi:10.2196/13782)
Supplement: Multimedia Appendix 1 [file periop_v3i1e13782_app1.docx]

Multimedia Appendix 1: PRISMA Flow Diagram

Records identified through database searching

(=2028)

Records after filtered by age, 19 years old and older

(n=736)

Records after filtered by year, 2008-present

(n=554)

Records excluded

(n=187)

Records included in literature review

(n=13)

Records screened

(n=200)

Records after filtered by language-English and full text

(n=519)
