# Supplementary material for: Impact of Intensive Care Unit Readmissions on Patient Outcomes and the Evaluation of the National Early Warning Score to Prevent Readmissions: Literature Review
Source: JMIR Perioper Med. 2020 May 8;3(1):e13782. doi: 10.2196/13782 (PMC7709858; doi:10.2196/13782)
Supplement: Multimedia Appendix 2 [file periop_v3i1e13782_app2.docx]

Multimedia Appendix 2: Article Tables

| **Citation** | **Purpose**  **Aims**  **Objectives** | **Study Design/**  **Method** | **Study Sample/**  **Setting** | **Major Variables (outcome variables)** | **Measurement of variables** | **Analysis** | **Results/**  **Findings** | **Quality of the**  **Evidence** |
| --- | --- | --- | --- | --- | --- | --- | --- | --- |
| Christmas, 2014 | To assess the most common events leading to RT* in trauma patients | RCR* of ICU* RT’s from 2004 to 2008 at an 874 bed Level I trauma center; utilized the trauma registry for data collection | 158 Pts* reviewed  874 bed Level I trauma center  Inclusion:  Initial admission to the ICU required  Exclusion:  -RT after planned surgical procedure  -Pts not surviving initial ICU admission | -ICU LOS*  -Injuries  --TBI**  --Rib fractures  --pulmonary contusion  -Injury severity  -demographics  -Interventions  Reason for RT:  -Cardiac  -Respiratory  -Neurologic  Required intubations on RT  Mortality | 158 RT’s (3.2%):  -Respiratory failure 48%  -Cardiac 16%  -Neurologic 13%  Most common injuries:  -TBI 32%  -Rib fractures 30%  -Pulmonary contusions 20%  Initial ICU LOS 6.6+-8 days  Ventilator days 4.4+-7.7 days  Out of ICU time before RT 5.5+-6.3 days  -Intubations on RT 49pts (31%)  -RT incurred additional ICU -LOS of 8+-8.5 days  -Ventilator days 5.2+-7.5  -Mortality after RT 10% | Retrospective review  Evaluation of pts requiring RT  Evaluation of patient diagnosis  Evaluation of intervention following RT | Respiratory compromise leading cause of RT  TBI pts most vulnerable  Pulmonary hygiene practice out of the ICU is beneficial  RT is costly  RT increases patient mortality | Level of evidence : VII, expert opinion  Strengths: Trauma population, identifies injuries most at risk  Weaknesses: One center review  Applicability: Project to be performed on surgical critical care and trauma population. Will be performing study in this center. |

**Legend**: RT- Return Transfer, RCR- Retrospective Chart Review, ICU- Intensive Care Unit, Pts- patients, LOS- Length of stay, TBI- Traumatic Brain Injury

| **Citation** | **Purpose**  **Aims**  **Objectives** | **Study Design/**  **Method** | **Study Sample/**  **Setting** | **Major Variables (outcome variables)** | **Measurement of variables** | **Analysis** | **Results/**  **Findings** | **Quality of the**  **Evidence** |
| --- | --- | --- | --- | --- | --- | --- | --- | --- |
| Downey, 2017 | An attempt to summarize the strengths and weaknesses of the early warning score for patients and staff; the aim is to address the gap in the literature to guide improvements for the optimization of patient safety | Systematic Review and Narrative synthesis | Adult patients for which VS were collected  232 studies met inclusion criteria | Evaluation of strengths and limitations of the early warning score | Early warning score has good predictive value  An intermittent assessment—limiting its assessment ability | 825 papers identified in search  232 papers met inclusion criteria | Good predictive value  Better in some patient populations than others | Level of evidence : I  Strengths: Systematic review, looked at many studies and synthesized the data  Weaknesses: Broad search criteria was used in the literature review; not all the studies used were level I evidence  Applicability: Excellent review of the score for project, articles reviewed provided additional resources for review. |

**Legend:** VS- Vital Signs

| **Citation** | **Purpose**  **Aims**  **Objectives** | **Study Design/**  **Method** | **Study Sample/**  **Setting** | **Major Variables (outcome variables)** | **Measurement of variables** | **Analysis** | **Results/**  **Findings** | **Quality of the**  **Evidence** |
| --- | --- | --- | --- | --- | --- | --- | --- | --- |
| Fakhry, 2013 | Review ICU BB*  Define ICU discharge variables predictive of BB | RCR*  Data collected: 11/18/04 to 9/1/09  Review of trauma registry  All BB reviewed by two trauma surgeons | 88 pts* BB to ICU  -75% male  -25% female  Age 15 years and older  Excluded: -Return to ICU after planned surgery  -Death or BB 7 days or more from transfer  -DNR* order | -Pts returning to ICU or death within 7 days of discharge  -Pts gender  -discharge timing- day versus night shift  -Comorbidities  -Age  -Severity of injury | 1971 pts admitted to ICU and discharged alive  162 (8.2%) BB ICU or died after transfer | SAS Version 9.2  Descriptive statistics calculated for:  -Predictor variables  -Days to BB | -4.8% BB rate  -19.3% of BB associated with mortality  Strongest predictors of BB:  -Male  -GCS* <9  -High FiO2*  -Day shift discharge  -1 or > comorbidities  -Increased heart rate  (p <0.05) | Level of evidence : VII  Strengths: Evaluated many assessment criteria for reason for BB  Weaknesses: Review of one facility only; need a multi-institutional study for validation  Applicability: Looks at trauma population |

**Legend:** ICU- Intensive care unit, BB- Bouncebacks, RCR- Retrospective Chart Review, pts- patients, GCS- Glasgow Coma Scale, FiO2- Fractional inspiration of Oxygen, DNR- Do Not Resuscitate, ISS- Injury Severity Score

| **Citation** | **Purpose**  **Aims**  **Objectives** | **Study Design/**  **Method** | **Study Sample/**  **Setting** | **Major Variables (outcome variables)** | **Measurement of variables** | **Analysis** | **Results/**  **Findings** | **Quality of the**  **Evidence** |
| --- | --- | --- | --- | --- | --- | --- | --- | --- |
| Hosein, 2014 | Evaluation of the developed risk stratification tools and their ability to predict adverse outcomes for patients discharged from the ICU* | Systematic Review  Database review of the literature  Cohort studies selected that described derivation, validation or clinical impact of tools for predicting medical emergency team activation, ICU readmission or mortality following patient discharge from the ICU  Data extracted on study design, setting, population, sample size, tool, and outcomes | Studies including: Adult patients discharged from the ICU  Medical Surgical ICU  745,187 patients included  Age range 57-64 years | ICU Readmission  Post ICU mortality  Evaluation of risk assessment tool to predict ICU readmission | ICU readmission 2.1-8.3% among studies | 9926 articles found in initial search  With exclusion criteria applied 8 articles were included in the systematic review | 8 risk stratification tools evaluated  Limited comparative  Further evaluation of existing tools effects on care is required prior to clinical implementation | Level of evidence : I  Strengths: Systematic review, highest level of evidence  Weaknesses: Only eight of the 9000+studies found were reviewed in this case  Applicability: This study reviews the risk stratification tools being examined for implementation in this DNP project. |

**Legend**: ICU- Intensive Care Unit

| **Citation** | **Purpose**  **Aims**  **Objectives** | **Study Design/**  **Method** | **Study Sample/**  **Setting** | **Major Variables (outcome variables)** | **Measurement of variables** | **Analysis** | **Results/**  **Findings** | **Quality of the**  **Evidence** |
| --- | --- | --- | --- | --- | --- | --- | --- | --- |
| Johns, 2014 | To examine characteristics, risk factors, and outcomes of trauma patients with and without ICU readmission | Retrospective and descriptive study  Chart review of patients returning to the ICU* | 637 bed academic medical center  -Level 1 trauma center  -80 adult ICU beds  Trauma patients  -69.3% male  -67.6% white  -64.2% admitted from ED*  Inclusion:  -patient admitted with primary trauma diagnosis  -Admit 6/1/07 – 5/31/12  Exclusion  -Comfort care or DNR*  -D/c* from the ICU to a location where ICU readmission not possible  -Age <18 years  -Initial ICU stay <4 hours  -Organ donor  -Burn patients | -Patients readmitted to ICU  -Patients not readmitted to ICU | 900 trauma patients evaluated  -707 blunt injuries  -193 penetrating injuries | Retrospective review of patients readmitted to ICU and not readmitted to the ICU  Categorical data measured with the Fisher exact test  Continuous data variables assess with Mann-Whitney U test | Risk factors for readmit: Increased age, and diabetes  Low GCS*, low albumin level, and elevated glucose levels—show greater risk for readmit | Level of evidence : VI  Strengths: Large number of patients reviewed  Weaknesses: Single center  Applicability: Focus on the trauma patient which will be the focus of the quality improvement project; identifies patients at risk for readmission |

**Legend**: ICU- intensive care unit, DNR- Do Not Resuscitate, D/C-Discharged, ED- Emergency Department, Glasgow Coma Scale

| **Citation** | **Purpose**  **Aims**  **Objectives** | **Study Design/**  **Method** | **Study Sample/**  **Setting** | **Major Variables (outcome variables)** | **Measurement of variables** | **Analysis** | **Results/**  **Findings** | **Quality of the**  **Evidence** |
| --- | --- | --- | --- | --- | --- | --- | --- | --- |
| Kaben, 2008 | To evaluate the incidence of, outcome from, and possible risk factors for readmission to the SICU* | Prospective data analysis | 2852 patient’s d/c from SICU to floor  -1828 men (64.1%) | Readmission to the ICU  Number of times readmitted to the ICU  In hospital mortality rate | 476 readmissions  -223 were planned  -253 unplanned  Gastrointestinal surgery was the most common type of surgery performed on patients readmitted more than once  First readmission most related to cardiovascular incident  2^nd^ & 3rd readmission most related to gastrointestinal or pulmonary issues | Factors associated with readmission:  -Age, CI 1.03-1.24  -Sequential organ failure score, CI 1.01-1.08  -C-reactive protein on day of transfer, CI 1.01-1.04 | Readmission rate: 13.4%  -314 (82.4%)readmitted once  -39 (10.2%) readmitted twice  -28 (7.3%)readmitted more than twice  First readmission- median 7 days (5-14)  In hospital mortality for readmitted patient 17.1%, higher than other patients | Level of evidence : II  Strengths: Large study group  Weaknesses: Given this was observational it was unclear whether readmissions were appropriate or note  Applicability: DNP project will be performed in the surgical trauma ICU—same patient population |

**Legend**: SICU- Surgical Intensive Care Unit, d/c- discharged, ICU- intensive care unit

| **Citation** | **Purpose**  **Aims**  **Objectives** | **Study Design/**  **Method** | **Study Sample/**  **Setting** | **Major Variables (outcome variables)** | **Measurement of variables** | **Analysis** | **Results/**  **Findings** | **Quality of the**  **Evidence** |
| --- | --- | --- | --- | --- | --- | --- | --- | --- |
| Kareliusson, 2015 | Evaluate the SWIFT* score a predictor for readmission | Retrospective cohort study  -Data collected on all ICU admissions  -SWIFT score calculated | Tertiary teaching hospital, 600 beds  -8 bed ICU*  ICU length of stay: 3.7 days  Inclusion:  -Patients admitted to ICU  -Adult  Exclusion  -<18 years old  -post-op patient | -readmitted patients  -ICU mortality  -Mortality after 30 days  -Discharge with a SWIFT >15 compared to a SWIFT <15 | 1244 patients  -563 surgical  -458 medical  -46 oncology | Data comparison with the Student’s t test  Categorical data compared with Pearson’s squared test  STATA version 12.1 and SPSS version 20 | Readmission resulted in:  -increased ICU length of stay  -Increased ICU mortality  -Increased 30-day mortality  -higher with SWIFT >15 | Level of evidence : VII  Strengths: Looks at two different patient populations  Weaknesses: Single center study, Small ICU only 8 beds  Applicability: Identifies risk factors/predictors for ICU readmission, looks at medical and surgical patients |

**Legend**: ICU- Intensive Care Unit, SWIFT- Stability and Workload index for transfer

| **Citation** | **Purpose**  **Aims**  **Objectives** | **Study Design/**  **Method** | **Study Sample/**  **Setting** | **Major Variables (outcome variables)** | **Measurement of variables** | **Analysis** | **Results/**  **Findings** | **Quality of the**  **Evidence** |
| --- | --- | --- | --- | --- | --- | --- | --- | --- |
| Lee, 2009 | Evaluation of factors associated with early readmission to the ICU* during the same hospitalization and factors associated with adverse outcomes | Prospective observational study | 25,717 admits  -378 (1.5%) readmits within 3 days  -374 patients’ medical records available for review  2-year period of time  Age mean 61.3 yrs. | Most common cause of readmit to the ICU  Adverse outcomes associated with readmission | Cause of readmission:  -Respiratory 118 (31.6%)  -Cardiovascular 91 (24.3%)  Increased mortality when mechanical ventilation required (p<0.001) | Statistical analysis  SPSS software used  Student t test, Wilcoxon rank sum, and Kruskal-Wallis test were used | First ICU admission most related to cardiovascular  Readmission most related to respiratory and then cardiovascular | Level of evidence : II  Strengths: Large patient population  Weaknesses: Only early readmissions evaluated, unable to predict factors associated with readmission  Applicability: Supports that respiratory is a leading cause of ICU readmission. |

**Legend**: ICU- intensive care unit

| **Citation** | **Purpose**  **Aims**  **Objectives** | **Study Design/**  **Method** | **Study Sample/**  **Setting** | **Major Variables (outcome variables)** | **Measurement of variables** | **Analysis** | **Results/**  **Findings** | **Quality of the**  **Evidence** |
| --- | --- | --- | --- | --- | --- | --- | --- | --- |
| Martin, 2018 | Evaluate predictors of readmission among a diverse population of surgical patients and develop an accurate and clinically applicable nomogram for prospective risk prediction | Retrospective chart review  Prospective analysis | SICU pts  24 bed ICU, tertiary care medical center  Level 1 trauma center  Exclusion  -Planned readmit  -Death during initial admission  -transfer to outside facility  -d/c from hospital within 72 hrs of ICU d/c | 72-hour ICU readmission | Variables measured to predict readmission:  -demographic characteristics  -Preadmission comorbidities  -ICU admission diagnosis  -Lab values  -Vital signs | Wilcoxon rank sums test for nonparametric data  X2 and Fisher Exact test for categorical variables  Hanley and McNeil test  R software version 3.1.2 | 3,109 pts used for development of predictive model  ICU readmission occurred 5% (141 pts)  Reason for readmission:  -33% respiratory  -22% CV  -15% bleeding  -12% postoperative  Readmission mortality  -5% (7/141) | Level of evidence : III  Strengths: Model development based on patient population  Weaknesses: Single center  Applicability: SICU patient focused. |

**Legend**: ICU- intensive care unit, SICU- surgical ICU, CV: Cardiovascular, pts: patients

| **Citation** | **Purpose**  **Aims**  **Objectives** | **Study Design/**  **Method** | **Study Sample/**  **Setting** | **Major Variables (outcome variables)** | **Measurement of variables** | **Analysis** | **Results/**  **Findings** | **Quality of the**  **Evidence** |
| --- | --- | --- | --- | --- | --- | --- | --- | --- |
| Smith, G.B., 2013 | Evaluation of NEWS* to discriminate patients at risk of cardiac arrest, unanticipated ICU* admission, or death within 24 hours of a NEWS evaluation | Prospective observational study | 35,585 patients  Median age: 67.7  -Male 65.9 yrs., female 69.4 yrs.  198,755 vital sign data set reviewed | Vital signs  Observations that followed obtaining vital signs | Of the 198,755 data sets:  -199 followed by cardiac arrest  -1161 unanticipated ICU admission  -1789 by death  -3149 by any of the outcomes  (all within 24 hours) | Microsoft visual FoxPro 9.0 | 95% CI for NEWS for cardia arrest 0.722, unanticipated ICU admission 0.857, death 0.894, and any other outcome 0.873 | Level of evidence : II  Strengths: Vital signs were collected the same way every time  Weaknesses: Excluded patient admitted directly to the ICU, but did not exclude patients with do not resuscitate order  Applicability: Evaluates the use of the NEWS which will be implemented in the project |

**Legend**: NEWS- National Early Warning Score, ICU- intensive care unit

| **Citation** | **Purpose**  **Aims**  **Objectives** | **Study Design/**  **Method** | **Study Sample/**  **Setting** | **Major Variables (outcome variables)** | **Measurement of variables** | **Analysis** | **Results/**  **Findings** | **Quality of the**  **Evidence** |
| --- | --- | --- | --- | --- | --- | --- | --- | --- |
| Uppanisakorn, 2018 | To determine the ability of the NEWS* at ICU* discharge to predict the development of clinical deterioration within 24 hours | Prospective observational study | 440 patients  -mean age: 61 yrs.  -219 male  53.2% planned ICU discharge  46.8% unplanned ICU discharge | NEWS score prior to ICU transfer  Early clinical deterioration | Early clinical deterioration:  -CI 0.89-0.94, p<0.001  NEWS >7  -sensitivity of 93.6%  -Specificity of 82.2% | Continuous variable expressed in mean +- standard deviation  Chi squared test  Fishers exact test  Mann Whitney test | NEWS score of >7 at ICU discharge represented a better sensitivity and specificity to detect early clinical deterioration 24 hours after ICU discharge | Level of evidence : II  Strengths: NEWS calculated prior to every transfer, defined clinical deterioration  Weaknesses: Low number of cases reviewed  Applicability: Looks at the use of NEWS for prediction of ICU readmission which will be implemented in project |

**Legend**: NEWS- National Early Warning Score, ICU- Intensive Care Unit

| **Citation** | **Purpose**  **Aims**  **Objectives** | **Study Design/**  **Method** | **Study Sample/**  **Setting** | **Major Variables (outcome variables)** | **Measurement of variables** | **Analysis** | **Results/**  **Findings** | **Quality of the**  **Evidence** |
| --- | --- | --- | --- | --- | --- | --- | --- | --- |
| Utzolino, 2010 | Evaluating outcomes of patient in a SICU* with high daily turnover rates | Retrospective chart review | 2558 patients  20 bed SICU  1 year | SICU readmission rate- elective versus unplanned  Readmissions with surgical complications  Mortality rate with readmission  Reason for readmission | SICU readmission rate: -elective 139/1675 (8.3%)  -unplanned 110/439 (25.1%)  Overall readmission rate:  249/2114 (11.7%)  Readmissions with surgical complications:  125/249 (50%)  Mortality rate with readmission:  33/249 (13.3%)  Reason for readmission:  -Respiratory 44% risk of death | SAS 8.2 statistics software  T test  Fisher exact test | Discharge from the SICU to early results in higher readmission rates, increased mortality, if readmission associated with respiratory issue mortality risk is increased | Level of evidence : IV  Strengths: Large volume of patients over an extended time period  Weaknesses: Retrospective in nature no direct patient intervention  Applicability: Evaluates patients in the SICU which is the same setting as the project will be implemented |

**Legend**: SICU- Surgical Intensive Care Unit

| **Citation** | **Purpose**  **Aims**  **Objectives** | **Study Design/**  **Method** | **Study Sample/**  **Setting** | **Major Variables (outcome variables)** | **Measurement of variables** | **Analysis** | **Results/**  **Findings** | **Quality of the**  **Evidence** |
| --- | --- | --- | --- | --- | --- | --- | --- | --- |
| Ye, 2019 | Prospectively validate a real time EWS to predict patients at high risk for inpatient mortality during their hospital episodes | Retrospective and prospective cohort | Berkshire Health Systems Hospitals  January 2015 to September 2017  54,246 patients | Scored daily and long term risk for inpatient mortality | 99 encounters identified in high risk group, c-statistic 0.884  68/99 (69%) died during the episode  Accurately predicted possibility of death 34/255 (13.3%) at least 40.8 hrs before death | Tree based random forest method | Demonstrated capability of the newly designed EWS to identify high risk patient of in hospital death and alert clinicians.  Allowed opportunity for timely intervention  Assist in clinical decision making; more actionable, effective, individualized patient care | Level of Evidence: II  Strengths:  -Identifies the strengths of a screening tool in identifying high risk patients and assisting in clinician decision making.  Weaknesses:  -Observes the EWS where this paper is specifically evaluating the NEWS |

**Legend**: EWS- Early warning score, NEWS- National Early Warning Score
